# Buffering Academic Stress during the COVID-19 Pandemic Related Social Isolation: Grit and Growth Mindset as Protective Factors against the Impact of Loneliness

**DOI:** 10.1007/s41042-020-00043-7

**Published:** 2020-10-16

**Authors:** Magdalena Mosanya

**Affiliations:** 1 The Institute of Psychology of the Polish Academy of Sciences, Jaracza, 1, 00-378 Warsaw, Poland; 2 Middlesex University Dubai, Knowledge Park, Block 16, Al Soufuh 2, Dubai, United Arab Emirates

**Keywords:** Growth mindset, Grit, Loneliness, Academic stress, COVID-19

## Abstract

The pandemic of the SARS CoV-2 virus, which causes COVID-19 sickness, constitutes a global challenge to well-being. Positive psychology constructs of grit and growth mindset may offer a solution to this challenge as both are associated with psychological resilience. A growth mindset describes the underlying beliefs people have about the malleability of intelligence, and grit refers to dedication to long-term goals. The present study explored whether such constructs could constitute protective factors against the academic stress associated with loneliness and perceived lack of control among international students (*n* = 170) during social isolation, induced by COVID-19 restrictions. The results of a hierarchical multiple regression model explained 36% of the variance in academic stress with a perceived lack of control (*ß* = .53, *p* < .001) and growth mindset (*ß* = −.22, *p* < .001) being significant direct predictors. Moderation analysis explained 17% of the variance and confirmed that a level of dispositional grit moderated the detrimental influence loneliness had on academic stress. Simple slopes analysis revealed a significant effect for moderate (*β* = .07, *p* = .01) and high (*β =* .16, *p* = .001) levels of grit. Our findings suggest that grit and growth mindset, as dynamic variables, could be taught to students as resilience-building prevention of academic stress during the COVID-19 pandemic. Lastly, our results have shown that parents (37%) and friends (32%) were most frequently identified by students as supporters during the COVID-19 pandemic with minimal reported support from universities (2.5%).

By the end of 2019, COVID-19 began to spread around the world (Wu et al. 2020). Although most people infected did not experience severe symptoms, vulnerable individuals, the elderly, or with underlying medical conditions might develop a serious and life-threatening symptoms (World Health Organization (WHO) 2020). By March 11th, 2020, the WHO had declared COVID-19 spread a global pandemic and advised governments around the world to implement measures and restrictions to prevent its further spread. As the first preventive measures, virus screening and quarantine were implemented, as treatments or vaccines remained unavailable (Wu et al. 2020). Instead, physical distancing procedures were employed, as such efforts proved useful in tackling previous outbreaks of the SARS-1 in 2003 (Ferguson et al. (2006). Ferguson et al.As the number of cases rapidly increased globally and measures were enacted to isolate people from one another, people began to experience heightened anxiety, stress, (Galea et al. 2020). The impact has been felt globally, with some countries imposing stricter restrictions than others.

The United Arab Emirates (UAE) was the first Middle Eastern country to report a COVID-19 positive case, following the initial outbreak in China. Restrictions within the UAE were introduced in March 2020, with schools, universities, and other educational institutions ordered to close. As a result, educational providers began to offer online learning. During the months of social isolation and movement restrictions, the proliferation of cases was largely controlled, with comparatively few deaths reported in the UAE (WHO 2020). While necessary, such restrictions in social contact impeded much needed social support, the absence of which being is known to increase loneliness, stress, and a sense of vulnerability (Okruszek et al. 2020). During the initial stage of the epidemic in China, the psychological impact included moderate to severe levels of stress and anxiety (Wang et al. 2020). Negative consequences to well-being were reported in most severely affected countries (e.g., Brooks et al. 2020; Moccia et al. 2020; Okruszek et al. 2020; Zhang and Ma 2020). Recent reports on the impact of the COVID-19 pandemic on psychological health pointed anxiety and distress as the most common negative symptoms (Cao et al. 2020), with some factors like insecure attachment and anxious temperament elevating the risk of mental health disturbances (Moccia et al. 2020). Overall, it has been shown that the COVID-19 pandemic could lead to severe mental health problems (Fiorillo and Gorwood 2020). As pandemic-related stress was previously associated with significant long-term problems (Maunder et al. 2008), short- and long-term negative mental health consequences could result from pandemic-related social distancing and distress (Galea et al. 2020).

Stress can be defined as physiological arousal in response to environmental threats and challenges that are subjectively perceived as overwhelming an individual’s resources (Folkman and Lazarus 1991). It is a natural bodily reaction to challenge or demand;nonetheless, prolonged stress can diminish academic performance and provoke maladaptive behaviours (Vermunt and Steensma 2005). Furthermore, the consequences of stress are detrimental to the quality of life (Dusselier et al. 2005). The perception of stress stems from unpredictability and a perceived lack of control (Alsulami et al. 2018; Mineka and Kelly 1989). According to Jackson and Tessler’s (1984), a perceived lack of control is a function of adverse events. Within that theoretical perspective, academic stress might intensify during unexpected and dramatic external events like the COVID-19 pandemic, due to a lack of control and loneliness related to social isolation. Yet, more conclusive research is needed.

Social support is vital to mental health (Adams et al. 2016). According to the stress-buffering hypothesis model (Cohen and Wills 1985), social support attenuates the effect of adverse events and decrease stress and symptoms of depression among students (Musumari et al. 2018). Stress intensifies with lack of social support, isolation, and loneliness (Wang et al. 2020). Loneliness reflects perceived deficiencies in the quantity, quality, or type of relationships with others, which can lead to experiencing negative emotions and may affect one individual’s mental and physical health (Russell and Pang 2016). If imposed over a prolonged period, in the form of social isolation, such as seen during the COVID-19 pandemic, loneliness may lead to emotional separation, emotional pain, and impaired cognitive abilities, which can further be detrimental to one’s performance and health (Ditommaso et al. 2004; Goodwin et al. 2001). Cao et al. (2020) revealed a negative correlation of social support with pandemic-related anxiety among students in China. During the COVID-19 pandemic, individuals reported increased support from friends and family members (Zhang and Ma 2020). Such support relies mostly on sharing feelings and caring about family members and others. Furthermore, students living with parents were less likely to develop pandemic related anxiety (Cao et al. 2020). Interestingly, Maunder et al. (2008) highlighted the importance of institutional support in times of pandemic, suggesting that universities could play an essential role in assisting students. No studies on the perception of support have been done so far on students during the COVID-19 pandemic, so exploration of that topic is due.

## Theoretical Framework

Studies on medical personnel during past epidemics revealed that reducing pandemic-related stress and loneliness may be best accomplished through interventions designed to enhance resilience (Maunder et al. 2008). Positive psychology is a broad scientific discipline that focuses on exploring factors that facilitate well-being and resilience (Seligman and Csikszentmihalyi 2000), with grit and growth mindset as central notions. Resilience is referred to “as any response to academic or social demands that is positive and beneficial to development, such as seeking new strategies or putting forth greater effort” (Yeager and Dweck 2012, p. 303). In the context of external adversities, resilience might relate to the ability to dealing with distress and lack of control (Fletcher and Sarkar 2012). Hence, resilience is an invaluable psychological characteristic to develop, and growth mindset and grit as intertwined concepts have been shown to support resilient thinking and attitude (Duckworth et al. 2007).

A growth mindset**,** introduced as Implicit Intelligence Theory (Dweck 2007), can be explained as the belief “that basic qualities are things you can cultivate through your efforts” (Dweck 2007, p. 6–7). Blackwell et al. (2007) explained that when faced with challenges, individuals apply different strategies, otherwise called theories of intelligence. According to Dweck (2007), there are two theories: *incremental theory*, which is concerned with making progress, where setbacks are anticipated, and the *entity theory,* which involves reliance on innate ability and avoidance of challenges. These two approaches create different psychological worlds for students: first - a growth mindset, that tends to promote resilience, and second - a fixed mindset that endorses stagnation (Yeager and Dweck 2012). Substantial evidence indicates that mindset has an impact on learning, motivation, resilience, and performance (Burnette et al. 2012; Dweck 2007, 2012; Dweck and Yeager 2019).

A growth mindset is a known predictor of achievement, as students characterized by it exert more effort, try new strategies, and seek assistance when needed (Claro et al. 2016). Improvements in growth mindset also decrease academic stress and worry (Elliot and Dweck 2005; Yeager et al. 2016) and temper adverse effects of environmental factors like poverty on achievement (Claro et al. 2016). Although some have questioned the impact of mindset on students’ attainment (Li and Bates 2019), the meta-analysis and most recent randomized trials support a general positive effect of growth mindset on accomplishments and well-being (Burnette et al. 2012; Dweck and Yeager 2019; Yeager and Dweck 2012). The theoretical framework proposed by Burnette et al. (2012) suggested that such an impact is due to mindset influence on self-regulatory processes, which, in turn, predict goal achievement. Furthermore, a growth mindset is a dynamic quality, and a large body of research evidenced that interventions can be effective in teaching people incremental strategies (Broda et al. 2018; Claro et al. 2016; Dweck and Yeager 2019). Growth mindset could arguably be supportive of academic well-being and attainment during stressful times of social isolation related to measures undertaken to fight the spread of the COVID-19. Furthermore, interventions targeting mindset could be supportive of resilience-building during the pandemic.

Grit, within a positive psychology paradigm, involves passion and perseverance (Duckworth 2016), mental durability in striving towards accomplishments (Reed and Jeremiah 2017), is a non-cognitive skill that has shown to highly predict achievement (Alan et al. 2019; Datu et al. 2018). Grit refers to a student’s ability to persist after setbacks with the research identifying a positive impact of grit on determination, self-control, and self-regulation (Duckworth et al. 2007). Besides, grit positively influences mental health via association with lower stress, depression, and anxiety (Mosanya 2019; Jin and Kim 2017; Musumari et al. 2018; Zhang et al. 2018), and augmented positive emotions (Datu and Restubog 2020).

**Fig. 1:**
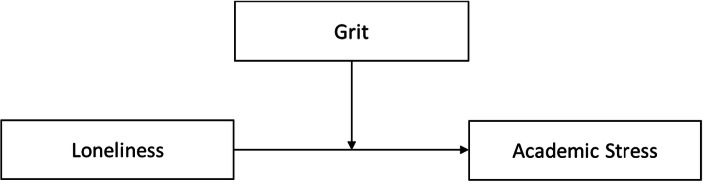
Conceptual model of grit as moderator of the relationships between loneliness and academic stress based on Hayes (2013)

So far, we have presented supportive evidence of grit’s direct impact on well-being and achievement. Grit has also been explored as a moderator of the relationship between adverse external events and internal states, like emotions and behaviours (Blalock et al. 2015; Kabat-Farr et al. 2019; Moles et al. 2017). Moreover, a higher level of dispositional grit buffers against the harmful effects of negative self-beliefs on athletic performance (Moles et al. 2017). Such a moderation effect has been explained in a way that perseverance in improving skills and maintaining passion neutralizes and/or diminishesperformance anxiety related to negative self-evaluations. Evidence from literature, therefore, motivates further exploration of the potential buffering role of grit on the loneliness-academic stress relationship during the COVID-19 pandemic. Figure 1 presents the suggested moderation model.

## International Students in Times of a Pandemic

The international student population in the UAE reflects the country’s multiculturally diverse social structure (Nickerson 2015). International students, exposed to different cultural paradigms and perhaps disconnected from their typical social supports may neither relate to their parents’ culture or the native culture of the UAE (Dillon and Ali 2019). In literature, ‘*transculturalism’* has been associated with lowered levels of psychological well-being, success, and integration (Nguyen et al. 2009; Wertsch 2004), placing multicultural individuals at elevated risk of developing stress and loneliness-related mental issues.

The risk of mental health disorders, including psychological distress, is, in general, high among university students (Farrer et al. 2016; Fawzy and Hamed 2017) and might increase with a lack of social support for international students (Cao et al. 2020; Wang et al. 2020). In addition to limited social assistance, online learning imposed on educational institutions during the COVID-19 pandemic could lessen human interactions and peer/teacher support, in turn decreasing the institutional impact on stress prevention. The psychological effects of COVID-19 could hence be particularly severe on international students and would justify further research on resilience-building among this population. It is recommended that universities respond to a mental health emergency and continue to develop means to improve student well-being and attainment (Zhai and Du 2020). Additionally, there is a significant gap in research on the psychological well-being of multicultural individuals (Syed and Juang 2014). Addressing the specificity of international students is therefore timely, as they might be at increased risk of developing COVID-19 related academic stress.

## Aims

Recent esearch has identified the adverse impact of epidemic-related loneliness and lack of control on mental health (Cao et al. 2020; Galea et al. 2020; Moccia et al. 2020; Okruszek et al. 2020). Hence, there is a need for interventions based on psychological factors that could support resilience during the COVID-19 pandemic (Maunder et al. 2008; Shanahan et al. 2020), and growth mindset and grit are central notions linked to resilience among students (Duckworth et al. 2007). The theoretical overview demonstrated strong support for the role that growth mindset and grit have in resilience building, and we suggest their direct impact on academic stress.

Thus, the present study first aimed to model the positive effects of loneliness, perceived lack of control, and negative effects of growth mindset, and grit on academic stress, as little research has been conducted to address the intersection of these concepts. As grit is known to buffer against negative experiences (Blalock et al. 2015; Kabat-Farr et al. 2019), the second aim was to assess grit’s moderating role in the positive relationship between loneliness and academic stress for international students socially isolated due to COVID-19.

## Methods

### Participants

In total, 170 undergraduate international students at a British overseas university were recruited via purposive sampling, as the objective of the study was focused on multicultural individuals. The sample consisted of 145 females (86%) and 25 males (14%) with a mean age of 21.74 years (*SD* = 6.7); ages ranged from 16 to 56 years. Participants were international students from 32 countries, with the majority being from India (45%), Pakistan (8%), and Great Britain (4%). All participants were fluent in English, a requirement for university admission. Approximately 105 (65%) participants classified themselves as having grown up as Third Culture Kids based on a given definition of having spent a significant part of their developmental years (under 18) outside either of their parent’s country of origin or their own (Pollock and Van Reken 2009).

### Procedure

The university’s Research Ethics Committee granted ethical approval. Students were approached via email, and data were collected online using Google Forms in April 2020, during a period of stringent lockdown measures in the emirate of Dubai. Firstly, respondents were given information about the study concerning the COVID-19 context with a statement*: You are being invited to take part in a research study. This study aims to find out protective factors to stress and loneliness during the COVID − 19 imposed social isolation measures, taking a positive psychology perspective.* Participants provided informed consent before taking part in the study.

### Measures

All scales were administered in English. A demographic questionnaire collected information on sex, age, passport country, and multiculturality. Supportive factors were assessed qualitatively with the question*:* Who/what has been of best support to you in recent times of social isolation related to measures limiting the spread of COVID-19?

*The Academic Stress Scale* was adapted from the University Student Stress Scale (Burge 2009). The scale validity was presented in the study of Azila-Gbettor et al. (2015). Items were rated on a 7-point Likert-type scale that ranged from 1 (*strongly disagree*) to 7 (*strongly agree*). The questions were modified to match online learning with an instruction: Please indicate how stressful you are for the following aspects of online academic work during the COVID-19 related social isolation and online learning - sample item: Studying for online tests and exams. For the current study, this scale had Cronbach’s alpha of .81.

*The Implicit Theories of Intelligence Scale* (Dweck 1999) is an 8-item growth mindset scale that assesses students’ beliefs whether their level of intelligence is fixed or malleable. Items were rated on a 6-point Likert-type scale that ranged from 1 (*disagree a lot)* to 6 (*agree a lot*). Its discriminant validity has been shown by Dweck et al. (1995). A sample item was: No matter how much intelligence you have, you can always change it a good deal. For the present study, the Cronbach’s alpha was .60.

*The Grit Scale* (Duckworth and Quinn 2009) assesses trait-level perseverance and passion for long-term goals and consists of 12 items scored on a 5-point Likert scale that ranged from 1 (*very much like me*) to 5 (*not at all like me*). It has high internal consistency, test-retest stability, consensual validity with informant-report versions, and predictive validity (Duckworth and Quinn 2009; Mosanya 2019). A sample item: New ideas and projects sometimes distract me from previous ones. For the current study, Cronbach’s alpha was .76, suggesting good reliability.

*Perceived lack of control* was measured with selected items of The Perceived Stress Scale (Cohen and Williamson 1988) items designed to tap how unpredictable, uncontrollable, and overloaded respondents find their lives. The instruction was: Please, while answering the following questions, relate to last month and the influence the COVID-19 pandemic had on you. Items were rated on a 5-point Likert-type scale that ranged from 0 (*never*) to 4 (*very often*). The scale’s validity has been established in the study of Vallejo et al. (2018). Sample item: In the past month, how often have you felt that you were unable to control the important things in your life? For the present study, Cronbach’s Alpha was .89 suggesting excellent reliability.

*Loneliness Scale – Short* (Ditommaso and Spinner 1993) consisted of fifteen items selected from the original SELSA subscales. For the present study, only ten questions related to Social and Family Loneliness were included. Concurrent validity for the scale was presented in the study of Ditommaso et al. (2004). Items were rated on a 7-point Likert scale that ranged from 1 (*strongly disagree*) to 7 (*strongly agree*). Sample item: I don’t have any friends who share my views, but I wish I did. The internal reliability for the total scale was .87.

## Results

### Pearson’s Correlations and Descriptive Statistics

Table 1 presents the means, standard deviations, and pairwise Pearson’s correlation coefficient of each scale’s total scores (*see* Table 1). There were positive significant pairwise correlations between levels of academic stress with loneliness and perceived lack of control. Growth mindset and grit were both negatively associated with academic stress, and grit individually negatively correlated with perceived lack of control and loneliness.

**Table 1:** Means, Standard Deviations, and Pearson’s Correlation Coefficient for Growth Mindset, Grit, Academic Stress, Perceived Lack of Control, Loneliness

Variables	M (SD)	1.	2.	3.	4.	5.
1.Growth Mindset	27.23 (4.65)	1	.32***	−.25**	–	–
2.Grit	38.19 (7.17)	–	1	−.33***	−.37***	−.18*
3.Academic Stress	23.64 (5.78)	–	–	1	.56***	.20***
4.Lack of Control	23.40 (7.94)	–	–	–	1	.36***
5.Loneliness	26.33 (7.57)	–	–	–	–	1

*p < .05, **p < .01, and ***p < .001

### Hierarchical Multiple Regression Analyses

A hierarchical multiple regression analysis was performed (Cook’s D values <1) to investigate the effect of the loneliness on academic stress (*see* Table 2). From the model 1 summary, it can be asserted that loneliness accounted for 4% of the variation in academic stress. The model fit the data well, and loneliness was a negative predictor of academic stress. The next predictor, grit, was added to model 2. Loneliness and grit accounted for 13% of the variation in academic stress. Both loneliness and grit were significantly predicting academic stress, yet adding grit to the model pointedly decreased the significance of loneliness impact on academic stress suggesting an interaction between these two/three factors. Model 2 explained significantly more variance than model 1. Furthermore, the growth mindset and lack of control were entered into model 3. Loneliness, grit, growth mindset, and lack of control together accounted for 36% of the variation in academic stress. The model fit the data well, with an only growth mindset and lack of perceived control significantly impacting academic stress, with the latter being the most potent positive predictor. Model 3 explained the most variance out of the three models.

**Table 2:** Hierarchical Multiple Regression results of Loneliness, Grit, Growth Mindset, and a Lack of Control as predictors of Academic Stress

	Model 1			Model 2			Model 3		
Predictors	b	SE	β	b	SE	β	b	SE	β
Loneliness	0.10	.04	.20 **	0.08	.04	.15*	−0.02	.04	−.03
Grit				−0.25	.06	−.30**	−0.65	.06	−.08
Growth mindset							−0.25	.06	−.22**
Lack of control							0.36	.45	−.54**
R ^2^ Adj	.04			.13			0.36		
F	7.18 (1; 168)**			12.14 (2;167)**			22.05 (4;165)**		
F for change in R ^2^	.04**			.09**			.23 **		

β = standardized regression coefficient; *p < .05;** p < .001

### Moderation Analysis

Multiple regression analysis revealed the interactions between grit and loneliness on academic stress and motivated further investigation of the moderating effect of grit. The outcome variable for analysis was academic stress (DV). The moderation model was significant (*p* < .001) and explained 17% of the variance in academic stress (*R*^*2*^ = .168). The predictor variable for the analysis was loneliness (IV), and the moderated variable evaluated for the analysis was grit (IVM). The interaction between loneliness and grit was found to be statistically significant (*β* = .12, 95% CI [.003, .022], *p* = .01). The conditional effect of loneliness on academic stress has shown corresponding results. At (the) low moderation (31), the conditional effect was non-significant (95% CI [−.114, .088], *p* = .79). At (the) middle moderation (38), the conditional effect was statistically significant (*β* = .07, 95% CI [.001, .14], *p* = .03). At (the) high moderation (45), the effect was statistically significant (*β* = .16, 95% CI [.063, .261], *p* = .001). These results identified grit as a moderator of the relationship between loneliness and academic stress. Only moderate and high levels of grit had the buffering potential to change the impact loneliness had on academic stress.

### Support

Investigation of the participants’ answers related to ‘supportive factors’ during COVID-19 isolation revealed that most of the students (38%) indicated parents as the best support, followed by friends (33%), significant others (9%), self (8%), siblings (4%), Netflix (3.4%), and lastly, university (2.5%).

## Discussion

While steps taken to prevent the spread of the COVID-19, including lockdown, physical and social distancing, and curfew, may be critical to mitigating its spread, they might have negative consequences on mental health (Cao et al. 2020; Galea et al. 2020; Moccia et al. 2020). The results of the present study confirmed the proposed hypothesis about the effect of loneliness and perceived lack of control on academic stress for international students in the context of COVID-19 imposed social isolation. Additionally, the current research suggested that these relationships can be attenuated by factors related to the positive psychology paradigm, namely, growth mindset and grit. Such an outcome goes beyond previous research, pointing to the vital role of grit and growth mindset in stress prevention in an educational context.

Firstly, the proposed hierarchical multiple regression model was best explaining academic stress variance (36%) when loneliness, grit, growth mindset, and lack of control were included. All predictors individually affected academic stress, but while compiled in one model, only growth mindset and perceived lack of control shown to be significantly impacting academic stress suggesting intermediary effects. Perceived lack of control, unsurprisingly, was the strongest predictor. These results, hence, might indicate that academic distress is related to situational factors like the unpredictability of the COVID-19 pandemic (Mineka and Kelly 1989), and internal dispositions like growth mindset (Dweck 2007). As growth mindset was a direct negative predictor of academic stress, it can be concluded that growth mindset assists educational stress reduction, which is in line with previous studies (Elliot and Dweck 2005; Mosanya 2019). The impact on well-being may be explained by the fact that individuals with a growth mindset have better self-regulation (Burnette et al. 2013) and increased positive affect (Elliot and Dweck 2005), which might support resilience to stress during the pandemic. Furthermore, the perceived lack of control as a function of external adversities can significantly increase during social isolation (Jackson and Tessler 1984) and impact academic stress. Our study expands the understanding of such effect onto the educational context, in line with predictions (Galea et al. 2020; Okruszek et al. 2020) and experience from the previous pandemics (Maunder et al. 2003).

Secondly, grit and loneliness were individually related to academic stress first as a buffer, and second as an intensifier. These findings supported previous literature as grit was shown to allay adverse environmental effects (Blalock et al. 2015), and loneliness to increase distress (Goodwin et al. 2001). The stress-inducing effect of loneliness can be explained through the increase in experienced helplessness, as loneliness has been shown to reduce prosocial behaviours like seeking assistance, and in turn to exaggerate the experience of powerlessness and stress (Lunn et al. 2020). Our moderation analysis brought a comprehensive understanding of the relationship between grit and academic stress, with the level of grit buffering the negative impact that loneliness had on academic stress. These outcomes supported the view that students with moderate and high levels of grit would be less impacted by loneliness and would experience less helplessness-related stress due to social isolation during the COVID-19 pandemic. One explanation to such effect might be that grit, as shown to be related to help-seeking behaviours, supports students’ cognitive coping and search for support, which in turn decreases their helplessness, isolation, and, indirectly, academic stress (Karabenick 2003). Drawing on previous research, a high level of grit can hence be regarded as a protective shield from the adverse impact of COVID-19 pandemic on students (Blalock et al. 2015; Kabat-Farr et al. 2019; Vainio and Daukantaitė 2016). Our results thus add to the existing research a new standpoint within an academic context.

The last outcome of the present study was the frequency of the participants’ answers about supportive ‘others’ during COVID-19 social isolation. Social support constitutes a substantial factor in regaining psychological balance after high-stress events and often allows individuals to adapt to life circumstances (Jou and Fukada 2002). Parental support has been presented in previous meta-analyses as predictive of students’ achievement (Catsambis 2001; Fan and Chen 2001) and low level of stress and anxiety (Zimmerman et al. 2000). Similarly, social support from friends protected against the depressogenic effect of terrorism-related perceived stress (Shahar et al. 2009). Not surprisingly, in our study, family members and close friends have been recognized as essential in dealing with the COVID-19 situation, with most students pointing towards support from their parents (38%) and close friends (33%).

Interestingly, the university as an institution has not been seen as a source of considerable support (2.5%). Such results might suggest that educational institutions like universities could be more actively engaged in assisting students during the recent COVID-19 pandemic as institutional support was shown as essential to preventing stress, as indicated by Maunder et al. (2008). Universities may need to extend beyond their purvey as providers of educations and work to enhance the psychological well-being of students.

Overall, it can be concluded that growth mindset and grit have supportive potential to buffering stress and loneliness during times of the COVID-19 pandemic. Experience from the epidemic of SARS-CoV-1 has demonstrated a need for resilience-building interventions (Maunder et al. 2008). Prophylaxis programs targeting a growth mindset and grit enhancement could be provided to students to increase their resilience and academic achievement (Paunesku et al. 2015; Datu et al. 2018). Dweck (2012), in her book *Self-Theories*, described the mechanisms of growth mindset and proposed successful techniques for developing it. Similarly, the impact of grit interventions suggests that grit is not only important but also that it is malleable and should be encouraged (Alan et al. 2019; Eskreis-Winkler 2015). We, therefore, propose that these two dynamic factors could be included in the necessary intervention programs during the pandemic of the COVID-19 delivered by universities.

### Limitation and Future Direction

Some limitations need to be considered when interpreting our results, inviting for further research. Firstly, our sample was limited, and participants were from diverse origins with a prevalence of South Asians. The potential influence of their cultural backgrounds on the results cannot be excluded as cultural factors shape stress response (Aldwin 2004). Also, purposive sampling limited the generalizability of the study findings. Furthermore, the gender distribution was not equal with more female participants, which might further decrease the generalizability of the current results, as females have shown to be more prompt to develop stress in previous pandemic studies (Xu et al. 2011). The cross-sectional, correlational nature of our research has made it impossible to establish real causal relationships and check for long-term associations. To account for these limitations, future research should replicate our results with different samples during an ongoing pandemic and replicate our results using longitudinal designs. Besides, it is vital to explore additional factors which could expand our model.

## Conclusions

By July 31st, 2020, COVID-19 has resulted in more than twenty million cases worldwide and 62,000 within the United Arab Emirates (WHO 2020), paralyzing life for many more. In a multicultural environment like the UAE, stress levels might be increased due to diversity and lack of social support for students (Yusoff 2012). Hence, international students upon whom social isolation was imposed as a preventive measure were at risk of developing mental health problems related to loneliness and stress. This study fills the gap in research on the effect of pandemic-related loneliness on stress in the academic context, highlighting its adverse impact. As some virologists forecast that global pandemics might become a recurrent phenomenon (Daddar and Nirupama 2015), prophylaxis of academic stress should include support of resilience reinforced at the organizational level (Maunder et al. 2008). Therefore, the present study proposes that universities provide targeted interventions to enhance the growth mindset and grit of their students.
